# Assessment of vaccine management performance in health facilities of Mwanza Region, Tanzania: a cross-sectional study

**DOI:** 10.1186/s40545-023-00651-z

**Published:** 2023-11-15

**Authors:** Green Sadru, Mulatedzi Makhado, Omary Swalehe, Stany Banzimana, Domina Asingizwe, Shital Mahindra Maru

**Affiliations:** 1 EAC Regional Centre of Excellence for Vaccines, Immunization and Health Supply Chain Management, College of Medicine and Health Sciences, University of Rwanda, Kigali, Rwanda; 2 Immunization and Vaccine Development (IVD), Ministry of Health, Dodoma, Tanzania; 3 Interactive Research and Development, Pretoria, South Africa; 4 Mzumbe University, Dar es Salaam, Tanzania; 5 Faculty of Health Sciences, University of Nairobi, Nairobi, Kenya

**Keywords:** Vaccine management, Health facilities, Staffing, Knowledge, Practices, Information technology, Tanzania

## Abstract

**Background:**

Effective Vaccine Management (EVM) initiative provides the platform needed to monitor and assess the vaccine supply chain system to identify strengths and weaknesses of the system at all levels and enhance the development of continuous improvement plan to strengthen the system. This study was conducted to determine the vaccine management performance in Health Facilities of Mwanza Region, Tanzania.

**Methods:**

This was a descriptive cross-sectional study that was carried out in 102 health facilities providing immunization services from eights districts of Mwanza Region in Tanzania. The World Health Organization (WHO) effective vaccine management assessment tools were used to collected data. Both quantitative and qualitative (through key informant interviews) approaches were used. The quantitative data were analysed using the existing WHO criteria for analysing effective vaccine management assessment data, while deductive thematic analysis was used for the qualitative data.

**Results:**

The finding shows that the overall score for vaccine management performance was 53% which is below the WHO acceptable minimum score of 80%. None of the health facilities had reached the benchmark but only 67% had an average performance (> = 50–< 80%). The highest health facility score was 76% and the lowest being 27%. Among the categories assessed, the highest score was on information technology with 72%, while the lowest was on standard operating procedures with a score of 43%. The major challenges which contributed to low performance were lack of training, low knowledge about vaccine management practices, unavailability of standard operating procedures (SOPs), and limited financial resources to support operations for vaccine management practices. Skills gap, incomplete stock records and management, as well as low availability of SOPs were the key challenges reported that affected vaccine management practices.

**Conclusions:**

Effective vaccine management performance was low across all districts under the study. Increasing personnel capacity and ensuring availability of resources to support operations were reported as key interventions in improving vaccine management practices. Hence, effectively working on continuous improvement plan with key highlighted actions is highly recommended to all actors from national level to sub-national level managers and healthcare workers as frontline vaccine handlers.

## Background

Immunization has proven to be a successful public health intervention and reducing effectively the under-five mortality rate [1]. In 2019, about 5.2 million deaths in under-five children occurred, most of which were caused by vaccine preventable diseases. Despite the proved impact of immunization, the World Health Organization (WHO) reported that vaccination coverage dropped from 86% in 2019 to 83% in 2020 globally [2]. One way to increase vaccination coverage is to ensure that vaccines are available when needed and are well-managed to maintain the potency. Effective vaccine management (EVM) is one of the initiatives that have been undertaken globally to ensure that potent vaccines reach the eligible population [1].

WHO and UNICEF with collective approach introduced a complete EVM framework in 2010 to help countries analyse the immunization supply chain performance and measure this performance against set standards. It assesses effectiveness and sufficiency of immunization supply chain (iSC) to identify weakness and shortcomings affecting the performance. It also measures the availability and quality of the six inputs categories on infrastructure, information technology, equipment, human resources, policies and procedures and financial resources as well as measuring availability, quality of outputs and performance [3].

Ensuring availability of high-quality vaccines is in every country's priority agenda, and effective vaccine management is required to maintain vaccine quality [4]. In addition, the immunization Agenda 2030 (IA2030), a global strategy to leave no one behind has set a focus on supply chains with focus to “Strengthen supply chains to ensure that high-quality vaccines are always available in the right quantity and form, at the right time, in the right place, stored and distributed under the right conditions” [5]. Ensuring right conditions requires effective vaccine management practices as recommended by WHO or country policies for each vaccine type.

One of the most important vaccine supply chain functions is keeping vaccines at recommended temperatures during transportation and storage [6]. WHO recommends vaccines to be stored between + 2 °C to + 8 °C to maintain vaccine potency and effective vaccine management practices scoring at least 80% [7]. Maintaining these recommended temperatures is challenging in practice, especially to areas with extreme hot or cold climates and unstable electricity [8]. Inappropriate vaccine management practices can contribute to increased vaccines wastage [9].

Knowledge and practices of vaccine handlers has effect on vaccine management practices but also subsequently on cold chain equipment (CCE) performance [4, 10]. The study in Cameroon found that vaccines stored in low temperatures was high in health facilities (51%) in health facilities as compared to 31% in district vaccines stores. Ineffective vaccine management practices increase risks of vaccine exposure to freeze or hot conditions that may lead to vaccine wastage but also breakdown of CCEs [11].

A study on cold chain management and vaccine stock management procedures to public health facilities in Ethiopia showed that most of the cold chain handlers (47.5%) had insufficient knowledge with significant number (51.2%) showing poor practices of cold chain management of the vaccines [6].

The lessons gained through the use of EVM assessments in more than 80 countries, utilizing developments in mobile and cloud-based computing informed the advancement to improve assessment and in 2019 another version was released. The new assessment tool expands on the previous version to give nations a wider, more effective, adaptable, and sustainable alternative for enhancing immunization supply chain [3].

The tool has nine (9) operational criteria, four (4) managerial criteria and 6 program management categories. The target score for each criteria and category at each level of the country's supply chain is set at a minimum of 80% required to be deemed acceptable practices. Each criterion is set to a number of set standards (requirements and sub-requirements) and scored out of 100% [3].

WHO recommends to countries conducting targeted assessment as a way to monitor progress on the implementation of continuous improvement plan following the EVM full national assessment. Tanzania conducted the EVM in 2015 in 2 primary stores (Tanzania mainland, Zanzibar), 23 regions, 28 districts and 56 health facilities. The overall score was 87% for 2015, which showed some improvement as compared to the previous EVMA in 2012, where the national score was 85% [12]. To enrich the improved EVM, Tanzania conducted an assessment in 2021 and had an overall score of 74% [13] which was below the bench mark of 80%.

However, less is known on what has been the root causes contributing to low vaccine management performance in health facilities providing immunization services in Tanzania. This served as the impetus for conducting this study as the targeted to determine the vaccine management performance and its associated factors in health facilities. The results of this study may guide national planning for continuous improvement of immunization supply chain.

## Methods

### Study setting

The research was conducted in selected immunizing health facilities in Mwanza region, Tanzania between December 2022 and January 2023. Mwanza region is one of the six regions in the Lake zone and it has a population of 3,699,872 with 595,680 children under-fives who were targeted for vaccinations, making it one of the highly populated regions in the country to administer vaccines [14]. It is one of the regions with diverse community with hard to reach areas that include islands, nomadic and urban population. It has a multicultural community due to its economy growing and being a hub for the lake zone. Administratively, Mwanza region has eight district councils and a total of 586 health facilities of which 360 health facilities were providing immunization services at the time of study.

### Study design

The facility-based descriptive cross-sectional study design using both quantitative and qualitative approaches was employed. The study through quantitative approach included both primary and secondary data collected to obtain data on vaccine management as per WHO/UNICEF recommended EVM Assessment Tool. The qualitative approach involved key informative interviews with district immunization managers to explore managers’ perceptions on vaccine management practices.

### Population and sampling

The Logistic Indicator Assessment Tool (LIAT) was used to determine the sampled health facilities needed for the quantitative study [15]. A sample size of 100 was obtained and 5% was added to accommodate non-response having a sample size of 106 out of the 360 health facilities providing immunization services. The study was conducted in all eight districts to get the sample in Mwanza region. The list of health facilities from each district were then selected based on the proportional size of the sample size. Then, through random sampling using the EVM assessment sampling tool (Table 1), the study units were obtained from each district in the region. The study was conducted in 102 out of 106 randomly sampled health facilities and the 4 remaining were not reachable due to geographical barriers following heavy rains and could not be accessed during the whole period of study.

**Table 1:** Random selection of sites in Mwanza, Tanzania

Site selection completion date	18/12/2022
Site selection performed by External Partner	Zainab Rida Berry
Confidence	85%
Precision	10%
Total active locations	360
Total locations excluded	0
Total locations selected	106
Total Lowest distribution	8
Total service points (HFs)	106
Location inclusion percentage	29.4%

The qualitative data collection was collected through district managers who were interviewed, giving a total of eight key informant interviews.

### Data collection instrument

#### EVM 2.0 tool was used to collect quantitative data

Five of the nine criteria (*E2—temperature management; E5—maintenance and repair; E6—stock management; E8—vaccine management and E9—waste management),* all four management functions and four of six categories *(C3—information technology; C4—human resources; C5—policies and procedures and C6—financial resources)* of the EVM assessment were included. The variables assessed were the operational inputs, i.e., human resource capacity (staffing, training and knowledge on vaccine management), information technology (data management), policies and procedures (standard operating procedures) and financial resources to support operations in vaccine management. This also included checking for the practices (outputs and performance) on the operational functions (Criteria), reviewing health facility documents. In addition, observations were made in the health facilities to assess the vaccine management practices as per EVM assessment tool.

Besides, an interview guide was developed to help obtain insights from district immunization managers as key informants on the challenges they face in implementing effective vaccine management operations in health facilities.

### Data collection procedures

Three Research assistants were trained on using the EVM assessment tool for 1 day before actual data collection commenced. The session was aimed to build understanding on procedures for data collection and using the tool to assess the requirements, doing observations, and reviewing the documents to fill the required field in the tool. The field use of EVM tool was pre-tested 1 week before the actual data collection from conveniently selected one district and four health facilities in Shinyanga Region. The actual data collection took place from December 2022 to January 2023.

For qualitative data, a face to face interviews using structured interview guides with themes on the EVM assessment were conducted to obtain insights for vaccine management practices at health facilities.

### Data management and data analysis

The completed data from EVM App was automatically stored in a web-based system and export was done to allow for additional analysis and correlations. EVM defines minimum requirements (standards) for each category, applicable to particular criteria. Each EVM requirement is given a weighting (1 or 5), depending on the supply chain level. EVM questions were used to assess whether a requirement has been met or not. If a requirement is met, it scores one (1) or five (5) and if it is not met, it scores zero (0). EVM scoring is then based on a multilayer aggregation of the requirement scores for categories, criteria and finally to the composite score for the EVM assessment. Non-applicable requirements are not scored and do not affect the composite scoring of the corresponding category and criterion. Hence, the scores range from 0% (terrible) to 100% (perfect); however, WHO acceptable minimum score is 80%, whereby any scores above or equal to 80% is Green Colour coded (good performing), while if the scores are 50% to below 80% is Yellow colour coded (average scores) and any score below 50% is Red colour coded (poor performance).

The qualitative part of the study employed a deductive thematic analysis approach. The audio recorded interviews were transcribed and translated from Kiswahili to English. The main steps that were followed included reading through the data, coding the data, generating categories and themes, and finally interpretation of data. The initial coding was done by the first author. The second and third authors read the first draft of initial codes and suggested some additions, hence a final list of codes, categories, and themes was made. Finally, the codes, categories, and themes were applied to corresponding quotes.

## Results

### Health facility characteristics

All districts councils in Mwanza region were targeted for the assessment from which service delivery points (health facilities providing immunization) were randomly sampled proportionally from each council. Among the health facilities assessed, few reported to have received supportive supervision and of those almost half had received written feedback to help referring to actions points and recommendation following supportive supervision (Table 2).

**Table 2:** Health facility characteristics

Characteristics	Category	Frequency, *n* (%)
Cadre (*n* = 102)	Registered Nurse	27 (26)
	Enrolled Nurse	52 (51)
	Clinical officer	10 (10)
	Assistant Clinical Officer	2 (2)
	Medical Attendants	11 (11)
Supportive supervision (*n* = 102)	Yes	39 (38)
	No	63 (62)
Written Feedback (*n* = 39)	Yes	20 (51)
	No	19 (49)

#### Facility operations and management consolidated results (Heat map)

EVM assessment results has shown that regional overall score at health facilities was 53% (Table 3). All categories had scored below 80% WHO benchmark. The score ranged from 72% for C3-information technology to 43% for C5-policies and procedures, which had the lowest score. Of the all criteria, only M3-supportive supervision of the eighty facility and operation management criteria exceeded the benchmark score of 80%. Waste management (E9), as one of the other criteria scored 68% and 15% was obtained for MI, the annual needs forecasting.

**Table 3:**
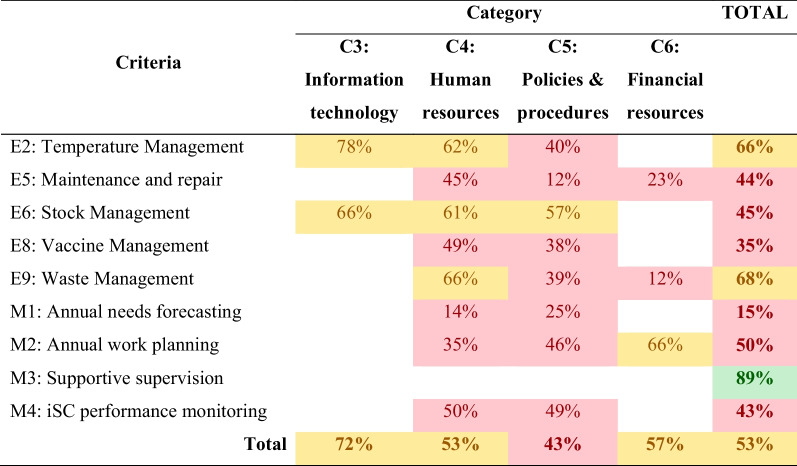
Facility operations and management consolidated results (Heat map)

The overall score on vaccine management was 53% and varied between health facilities with average highest score of 76% and lowest scores of 27% which are all below 80% WHO benchmark. Only 68 (67%) had at least reached the average score between 50% and 80% (Table 4).

**Table 4:** Health facility (HF) scores by district

District	HF highest and lowest score			Number and percent of health facilities per WHO Score range					
				> = 80–100%		> = 50–< 80%		< 50%	
	Highest (%)	Lowest (%)	Average (%)	*n* (%)	%	No	%	No	%
Buchosa	61	34	48	0	0	4	40	6	60
Ilemela	65	45	56	0	0	8	89	1	11
Kwimba	76	42	57	0	0	13	81	3	19
Magu	64	27	49	0	0	7	54	6	46
Misungwi	62	45	60	0	0	15	94	1	6
Nyamagana	73	42	58	0	0	14	93	1	7
Sengerema	59	35	48	0	0	5	42	7	58
Ukerewe	60	31	42	0	0	2	18	9	82
Total	76	27	53	0	0	68	67	34	33

**Fig. 1:**
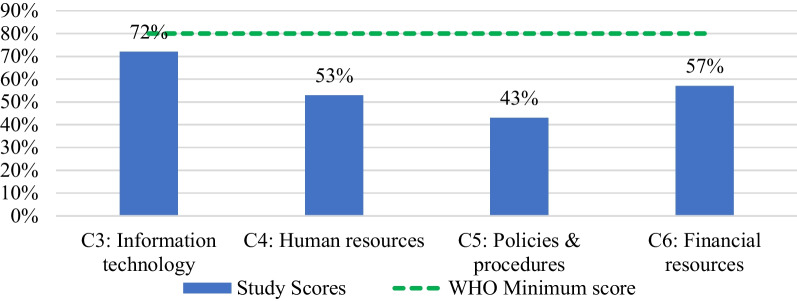
Overall EVM category scores of health facilities assessed, *n* = 102

The assessment conducted on the categories on health facility operations and management function which showed vitiations on performance and none of the category reached the WHO benchmark (Fig. 1).

#### Human resource capacity performance

The overall score was 53% for the human resource capacity with highest score of 66% on waste management followed by 62% on temperature management and the lowest scores was on annual needs forecasting with 14% (Table 5). The attributes for the human resources performance are described in Table 5.

**Table 5:** Human resources capacity on staffing, training and knowledge

	Frequency, *n* (%)
Staffing (*n* = 102)	
At least one member of staff is responsible for forecasting vaccine and dry goods needs	56 (55)
Adequate staff are assigned to monitor vaccine temperatures	60 (59)
At least one staff is assigned to carry out routine refrigeration maintenance tasks	35 (34)
Refrigeration technicians are available to maintain and repair CCEs	94 (94)
Two or more members of staff are assigned to manage vaccine stocks	57 (56)
Training	
How to forecast vaccine and dry goods needs	9 (9)
How to monitor vaccine temperatures	57 (56)
Routine refrigeration maintenance	77 (75)
Maintain an inventory of cold chain equipment	4 (4)
Vaccine stock management	58 (57)
Knowledge and understanding	
Key principles and procedures of temperature monitoring	72 (70)
Key principles and procedures of vaccine stock management	71 (70)
Key principles and procedures of vaccine management	50 (49)

*Staffing availability* The performance score on the staffing levels in most of the health facilities was below the WHO benchmark on the requirement to have at least one staff assigned to undertake various duties on vaccine management. The highest score that met the minimum acceptable level was on availability of technicians to support refrigerators maintenance from the district with 94 (94%) which met the recommended WHO score of 80% (Table 5). The availability of technicians was also reported through key informant interview (KII), where the District Immunization and Vaccines Officers (DIVOs) reported to support refrigerator maintenance upon being reported by the health facilities.

“*We normally receive the notification when the refrigerators get faults and we go to assess and fix what we find and when we fail then we report to high level……”* KII_03.

*Training* of staff responsible for vaccine handling and on monitoring vaccine temperatures had a score of 57 (56%), routine refrigerator maintenance 77 (75%) and vaccine stock management 58 (57%). However, most of the vaccine handlers reported to have not received training on vaccine and related supplies forecasting 9 (9%) and managing cold chain equipment inventory 4 (4%) (Table 5).

This was also discussed during KII. All district managers, reported that most vaccine handlers in health facilities have not received trainings. One stated:

“*In my District, most of healthcare workers taking care of the vaccines (vaccine handlers) have not received the training because they are either new or they have been re-located (staff rotation) from other departments or facilities and they only receive some skills when we go for supportive supervision*……” KII_06.

*Knowledge* It was reported that the knowledge on key principles and procedures of temperature management and vaccine stock management both had scored 70% and with least score on vaccine management 50 (49%) (Table 5). The same challenge was reported by all district managers that most of the vaccine handlers have low knowledge on vaccine management practices. One indicated:

*“It is challenging as the staff when they come in the RCH (reproductive and child health clinics) especially with vaccination services we find they have low knowledge on vaccine management and hence we find improper vaccine management and even the refrigerator is not well maintained as we expect ……...”* KII_05.

However, in temperature management most of the health facilities (HF) scored 90% to have not exposed vaccines to low damaging temperatures and only 76% reported to have not exposed vaccines to high temperatures.

### Health facility operations by EVM criteria

Effective vaccine management operations were assessed on various attributes for performance as a result of practices undertaken in vaccine management. Table 6 shows scores for the practices on each EVM criteria.

**Table 6:** Operational score on the criteria

	Frequency, *n* (%)
Temperature management, *n* = 102	
Vaccine storage temperatures are systematically monitored	45 (44)
Temperature alarms during storage are recorded and acknowledged	16 (16)
Temperature records are well-organized and secure	45 (44)
Vaccines are not exposed to damaging high temperatures during storage	78 (76)
Vaccines are not exposed to damaging low temperatures during storage	92 (90)
Maintenance and repair of CCE (*n* = 102)	
Cold chain equipment is maintained fully functional	79 (79)
Cold chain equipment is maintained in good physical condition	75 (75)
Cold chain equipment repair work is carried out promptly	92 (90)
Stock management (*n* = 102)	
Stock levels are documented for all vaccines	21 (20)
Stock levels are documented for all dry goods	15 (15)
Vaccine received are inspected and recorded upon arrival	39 (39)
Stock records are up-to-date	61 (60)
Vaccine stock records are complete	51 (50)
Vaccine stock records are well-organized and secure	50 (49)
Physical vaccine stocks counts are conducted regularly	24 (24)
Vaccine management (*n* = 48)	
Shake tests conducted in response to low temperature alarms	0 (0)
The correct diluents are used to reconstitute freeze-dried vaccines	7 (15)
Diluents are stored in the cold chain for at least 12 h prior to reconstitution	6 (13)
Opened multi-dose vials are marked with the date of opening	8 (17)

*On temperature management,* most of the health facilities scored 90% to have not exposed vaccines to low damaging temperatures but only 76% reported vaccines to have not been exposed to high temperatures, hence having a significant proportion of vaccines exposure to high temperature (Table 6).

*With maintenance and repair of CCE*, 92 (90%) of health facilities assessed reported to carry out repair for CCEs (Table 6).

Regarding stock management, most of the requirements had below 50% scores with the exception to stock records which had >  = 50% scores (Table 6).

*Looking at vaccine management,* despite of the health facilities being found with temperature alarms for low temperature that require to conduct shake test, none of the health facilities attempted to conduct a shake test and find if the vaccines can pass for use after exposure to freezing conditions (Table 6).

#### Information technology for vaccine data management

Three requirements out of six had scored above WHO benchmark. The available data management tools had scored above lowest acceptable WHO score with exception to vaccine stock record form (vaccine and related supplies ledgers) which had 51% and manual temperature monitoring form (Temperature monitoring charts) with 66% (Table 7).

**Table 7:** Information technology

*Data management technology*	Frequency, *n* (%)
The vaccine storage manual temperature monitoring form meets minimum requirements	67 (66)
Vaccine refrigerators have 30DTRs or equivalent	87 (85)
Vaccine refrigerators have backup temperature monitoring device	87 (85)
The vaccine stock record form has all of the required fields	53 (51)
The vaccine request form has all of the required fields	33 (83)
The vaccine receipt form has all of the required fields	82 (80)

The challenge on availability and use of updated data recording tools was also reported among district managers. One explained:

*“…for the past three years and more now, the program has not provided printed updated stock records……we have been trying to improvise while we wait for the supply of updated tools. And as I said, some have not been using properly the temperature monitoring charts”.* KII-08

#### Policies and procedures

Majority of the assessed health facilities missed the SOPs and hence the low scores of which the highest was 62% for vaccine vial monitor (VVM) followed by vaccine stock transactions. The lowest score was on SOPs for refrigerator maintenance which scored 12% (Table 8).

**Table 8:** Availability of standard operating procedures (SOPs)

Availability of SOPs for	Frequency, *n* (%)
Vaccine temperature monitoring in storage	41 (40)
Routine maintenance of refrigeration equipment	12 (12)
Managing vaccine stock transactions	57 (56)
Using Vaccine Vial Monitor (VVM)	63 (62)
Vaccine management	39 (38)
Immunization waste management	43 (42)

#### Vaccine management performance

**Fig. 2:**
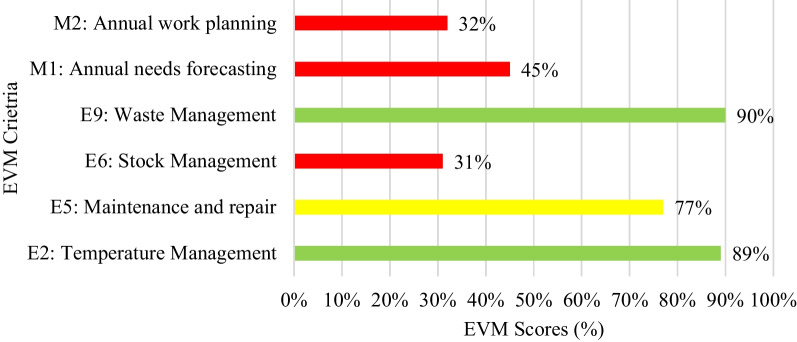
Vaccine management performance by criteria (*n* = 102)

The human resource capacity, availability and use of SOPs and data management tools as inputs categories to facilitate effective vaccine management practices have all met the WHO minimum standard for the temperature management and waste management. However, they had an average score for the maintenance and repair for not following the standard reporting procedures when the refrigerator need repair. The lowest scores were on stock management, annual forecasting and annual work planning score due to some of the records missed required fields, most of the health facilities had no vaccines forecasts and weak monitoring of workplans, respectively (Fig. 2).

## Discussion

This study assessed the vaccine management performance using EVM assessment tool established by WHO and UNICEF at health facilities. The overall score on vaccine management performance in Mwanza was 53%. Other assessments conducted in Tanzania had higher scores than this study [12, 13]. In addition, similar study conducted in Malawi found overall score of 87% [16] above the WHO benchmark of 80%.

### Vaccine handler capacity on vaccine management

The vaccine handler's capacity directly influences vaccines management procedures, and having supportive supervision helped them put their knowledge into practice. In this study it was found that only 38% of the respondents received supportive supervision [17]. This calls for attention to substantiate or motivate for the support of health facility workers to continue getting on job training.

The knowledge of staff responsible for vaccine handling is key to ensure effective vaccine management practices. It is anticipated that staff with knowledge will provide all necessary skills into recommended practices in the whole process of the immunization supply chain. In this study, the knowledge on key principles and procedures for vaccine management was below the minimum recommended WHO score. Other studies had also found lower scores [18–20]. Lower knowledge of staff handling vaccines increases risks to vaccines storage and ultimately administering vaccines of low quality to enable prevention of vaccine preventable diseases.

### Adherence to standard operating procedures on vaccine management

The standard operating procedures provide roles and responsibilities to be executed for vaccine management practices and hence being recommended as a requirement for each facility to have SOPs in place and attain the expected best practices. A study conducted in Cameroon showed that availability and use of SOPs had direct relations to vaccine management practices [11]. The study has shown that most of the health facilities lacked the SOPs and even those who had the SOPs, the practices did not show good performance to at least lead to scoring more than WHO benchmark. Effective use of SOPs has evidently helped to have common and recommended practices [22].

Inappropriate use of SOPs has a cause to exposed vaccines out of recommended range of + 2 °C to + 8 °C. Exposure of vaccines to high temperature was high compared to exposure to low temperatures in contrast with other studies found that vaccines were more exposed to low temperature (freeze excursion) than high temperature (heat excursion) [8]. Other studies have also shown that the vaccine handler knowledge and practices had highly contributed to the observed vaccines exposure to temperatures out of range [11, 21].

### Information technologies for vaccine management

The study found that majority of the health facilities (85%) had 30 DTRs to record continuous data on temperature. This is more than that was reported by WHO and UNICEF which had 48% health facilities with continuous temperature recorders [3]. This increases the opportunity to enhance effective temperature monitoring practices to maintain vaccine quality.

The effective vaccine management standard requires for each HF with vaccine in storage to have at least two staff assigned to monitor vaccine refrigerator temperature. However, in this study some of the health facilities had one staff assigned, where only 56% had at least two staff. Among the staff who were assigned, 56% had received training on temperature monitoring and have knowledge on key principles and procedures of temperature monitoring. The temperature management performance was low on monitoring the temperature alarms for immediate action.

Stock management practices score was low at 66% which was below the WHO benchmark. This is below the scores obtained in Ethiopia, where the score was 85.4%. The mostly contributing factors were lack of training leading to low knowledge to execute recommended practices on vaccine stock management [6]. Most of the health facility vaccine stock records did not have all required fields to capture all recommended vaccine information during storage and transactions. A study conducted in Ethiopia also found that only 46.3% stock records had complete information [6]

### Challenges facing vaccine management performance

The study also included key informant interviews with 8 district immunization managers to explore the challenges on vaccine management in health facilities. Two of the eight KIIs reported to have not received specific training on cold chain management, one of the key contributing factors on vaccine management practices.

Most of the HF reported to have not received funds as only 26% reported to have received funds to support cold chain maintenance. This was also reflected by KIIs who all responded to miss some operation funds specially to run the remote temperature monitoring systems and hence depending on the fridge tags hence a need to advocate for increased funding on allocating funds to support remote temperature monitoring (RTM) operations.

Through KII with district immunization managers it was reported that a delayed action following temperature hot or cold alarms has been supporting to provide feedback to track through web-based and notification [23]. Unstable power supply to run refrigerators in some of the health facilities were reported to have caused some of the health facilities having more faults.

Staff rotation has been an issue to maintain the skills impacted through the on the job training hence increasing the risk to delay on reporting the cold chain faults and performing daily, weekly and monthly tasks. The recommendations to ensure availability of staff trained on how to conduct maintenance and repair of refrigerators has been provided by different studies and standard guidelines [24]. Conducting training and implementing supportive supervision to conduct mentorship on sites was reported to be helpful in increasing capacity of the health facility staff to achieve effective vaccine management practices and performance. This was also recommendations to other studies [25].

Some health facilities were missed during assessment due to difficulties to reach and the 5% increase on the non-response rate increased the sample size and hence with 97% response rate. The study involved only 102 health facilities from one region out of 26 regions in Tanzania mainland hence the results may not be generalized. However, the study provides insights on knowledge and practices on vaccine management in Tanzania of which may be used to inform other regions for actions to improve vaccine management performance. This study suggests further research to explore more motivation and demotivation factors on use of SOPs to improve vaccine management practices.

## Conclusion

This study was conducted to determine the vaccine management performance and its associated factors in health facilities. The findings indicated that the effective vaccine management performance for health facilities in Mwanza region was low at 53% which is below WHO benchmark. None of the health facilities had reached the benchmark but only 67% had an average performance (> = 50–< 80%). This shows decreased availability of vaccines, quality of vaccines and efficiency of the immunization supply chain along with implementation. The low skills of assigned staff to handle vaccines increases the risks to vaccine management practices. Improving data management technology to capture all required information and raising knowledge and understanding on managing and handling vaccines with effective vaccine management practices is key towards improving immunization supply chain (iSC) performance.

## Data Availability

Data from this research is available upon request to the author.

## Abbreviations

CCE: Cold chain equipment
DIVO: District Immunization and Vaccines Officer
DTR: Daily Temperature Recorder
EAC: East African Community
EVM: Effective vaccine management
FT2: FridgeTag2
HF: Health facility
IA2030: Immunization Agenda 2030
iSC: Immunization supply chain
IVD: Immunization and Vaccine Development
KII: Key informant interview
LIAT: Logistic Indicators Assessment Tool
MoH: Ministry of Health
NBS: National Bureau Standards
NIMR: National Institute for Medical Research
PORALG: President’s Office for Regional Administration and Local Authorities
RTM: Remote Temperature Monitoring
SDD: Solar Direct Drive
SOPs: Standard operating procedures
UNICEF: United Nations Children's Fund
URT: United Republic of Tanzania
WHO: World Health Organization
